# Engineering high-efficiency matriptase substrates using *E. coli* display for applications in prodrug activation

**DOI:** 10.1016/j.crmeth.2025.101077

**Published:** 2025-06-10

**Authors:** Anna Mestre Borras, Hanna Mehari, Stefan Ståhl, John Löfblom

**Affiliations:** 1 Department of Protein Science, School of Engineering Sciences in Chemistry, Biotechnology and Health, KTH Royal Institute of Technology, 106 91 Stockholm, Sweden

**Keywords:** matriptase, tumor protease, protease substrate, prodrug, conditional activation, *E. coli* display, directed evolution, antibody engineering, protein engineering

## Abstract

Proteases play a crucial role in biological functions such as tumor progression and tissue homeostasis. Recently, protease-activated prodrugs have gained attention for their potential to enhance selectivity in tumor-targeted therapies. In this study, we report the engineering of substrate sequences for matriptase, a protease overexpressed in tumors and previously explored for prodrug activation *in vivo*. A peptide library containing millions of potential substrates was displayed on *Escherichia coli*, and flow cytometric sorting was used to isolate improved substrates based on cleavage efficiency. Hits were ranked by flow cytometry, and the top substrates exhibited k_cat_/*K*_M_ values over 40-fold higher than previously reported sequences. These substrates were further evaluated in an antibody-prodrug format, demonstrating exceptional activation. The matriptase substrates hold broad potential for applications such as cleavable linkers in next-generation antibody prodrugs. Furthermore, the developed bacterial display platform shows promise for discovering substrates of other proteases.

## Highlights

• High-throughput *E. coli* display enables screening of millions of protease substrates
• Top hits show >40-fold improved k_cat_/*K*_M_ over previously reported matriptase substrates
• Optimized substrates enable efficient antibody prodrug activation *in vitro*

## Motivation

On-target, off-tumor toxicity remains a challenge in targeted cancer therapy. Protease-activated prodrugs offer a strategy to increase selectivity by exploiting proteases enriched in the tumor microenvironment. However, their clinical success depends on the availability of highly efficient and specific protease substrates. In this study, we aimed to develop improved substrate sequences for the tumor-associated protease matriptase and to establish a generalizable method for discovering high-performance substrates. Such advances are valuable for enabling next-generation prodrugs with enhanced selectivity and safety.

## Graphical abstract

**Figure:**
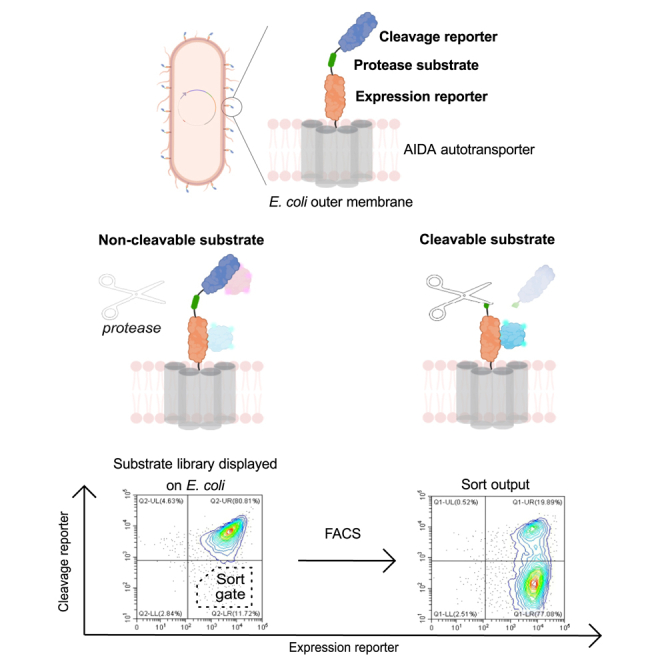

## Teaser

Mestre Borras et al. develop an *E. coli* display platform for high-throughput screening of protease substrates. Using this method, they identify matriptase-cleavable sequences that outperform existing substrates and enable efficient prodrug activation.

## Introduction

Proteases are critical components of many biological processes. In the context of tumors, their expression is frequently upregulated, which has been shown to promote tumor cell growth and invasion.^1^^,^^2^ Protease upregulation in the tumor microenvironment has also been explored for the development of next-generation cancer treatments. In the last decade, efforts have been made to develop drugs that are selectively activated in tumor tissues but remain inert in healthy tissues.^3^ Such constructs are commonly referred to as prodrugs, and their activation in the tissue of interest can be triggered by, for example, proteases in the tumor microenvironment.^4^ Several studies have been reported in which an antibody or another binding moiety is linked to a masking domain by a protease-sensitive linker. Proteases in the tumor environment act by unmasking the binder, thus allowing it to perform its function.^5^^,^^6^^,^^7^ Recently, we have reported on an affibody-based prodrug against epidermal growth factor receptor (EGFR) whose activation is triggered by matriptase.^8^^,^^9^^,^^10^^,^^11^^,^^12^

Matriptase, encoded by the ST14 gene, is a transmembrane type II serine protease found in epithelial tissues, where it is involved in epithelial barrier homeostasis.^13^^,^^14^ Its activity is tightly regulated by protease inhibitors such as hepatocyte growth factor activator inhibitor-1 (HAI-1).^15^ However, the expression of matriptase and its inhibitors is often dysregulated in tumor tissues, leading to an overactivation of the protease.^13^^,^^14^^,^^16^^,^^17^ Matriptase upregulation promotes cell invasion through extracellular matrix remodeling and activation of oncogenic pathways.^18^ Accordingly, its expression has been associated with many different types of cancers, including breast, colon, prostate, and lung, highlighting its role in cancer invasion and metastasis.^15^^,^^18^ Matriptase is composed of an extracellular domain where the active site is located, a transmembrane domain, and a cytoplasmic tail that anchors the protease to the actin cytoskeleton of the cell. The catalytic domain of matriptase is located in the active site and contains a catalytic triad formed by serine (nucleophile), aspartate (electrophile), and histidine (base).^19^ The geometric orientation of these amino acids is highly conserved in different serine proteases; thus, it is the size, structure, and charge distribution of the binding domain in the active site pocket that define the substrate specificity. Previous studies have shown that matriptase has a preference for substrates containing a positively charged amino acid in the position preceding the hydrolyzed peptide bond (P1). This specificity is conferred by a conserved negatively charged aspartic acid at the bottom of the binding pocket in the active enzyme.^19^^,^^20^

The aim of this study was to identify new matriptase substrate sequences that could be cleaved more efficiently compared to previously reported substrates.^5^^,^^20^^,^^21^^,^^22^ Related to prodrugs, such improved substrates would have the potential to increase the prodrug activation rate in the tumor tissue and hence increase its potency. Traditionally, the discovery of new protease substrates has often been achieved by using solution-phase fluorogenic substrate libraries. However, this method has a relatively low throughput, and the kinetic characterization of substrates becomes a bottleneck, as each peptide must be synthesized in sufficient quantities. To address this, we modified a previously described bacterial display method for screening and selection of matriptase-cleavable peptide substrates.^23^^,^^24^ The display method is based on the expression of recombinant proteins on the outer membrane of *Escherichia coli* using a genetic fusion to the autotransporter AIDA-I (adhesin involved in diffuse adherence).^25^ This method has previously been used for display and directed evolution of various affinity proteins, such as antibody fragments and affibody molecules.^24^^,^^26^^,^^27^^,^^28^ Here, the *E. coli* method was modified to display a designed combinatorial peptide library containing more than 4 million substrate variants between two reporter tags. The bacterial-displayed library was treated with matriptase, followed by the isolation of clones displaying cleavable substrates using fluorescence-activated cell sorting (FACS). The multivalent display on bacterial cells enabled high-throughput analysis of protease activity and allowed for the ranking of substrates based on their relative cleavage efficiency. The results were later compared to an established kinetic evaluation of soluble substrates based on Förster resonance energy transfer (FRET), demonstrating that the new peptides were cleaved much more efficiently (over 40-fold) than a previously reported matriptase reference substrate.^5^ Finally, the most promising substrates were tested in an antibody prodrug format, confirming their capacity for prodrug activation upon matriptase treatment *in vitro*.

## Results

### An *E. coli* display-based high-throughput method enables the screening of protease substrates

**Figure 1:**
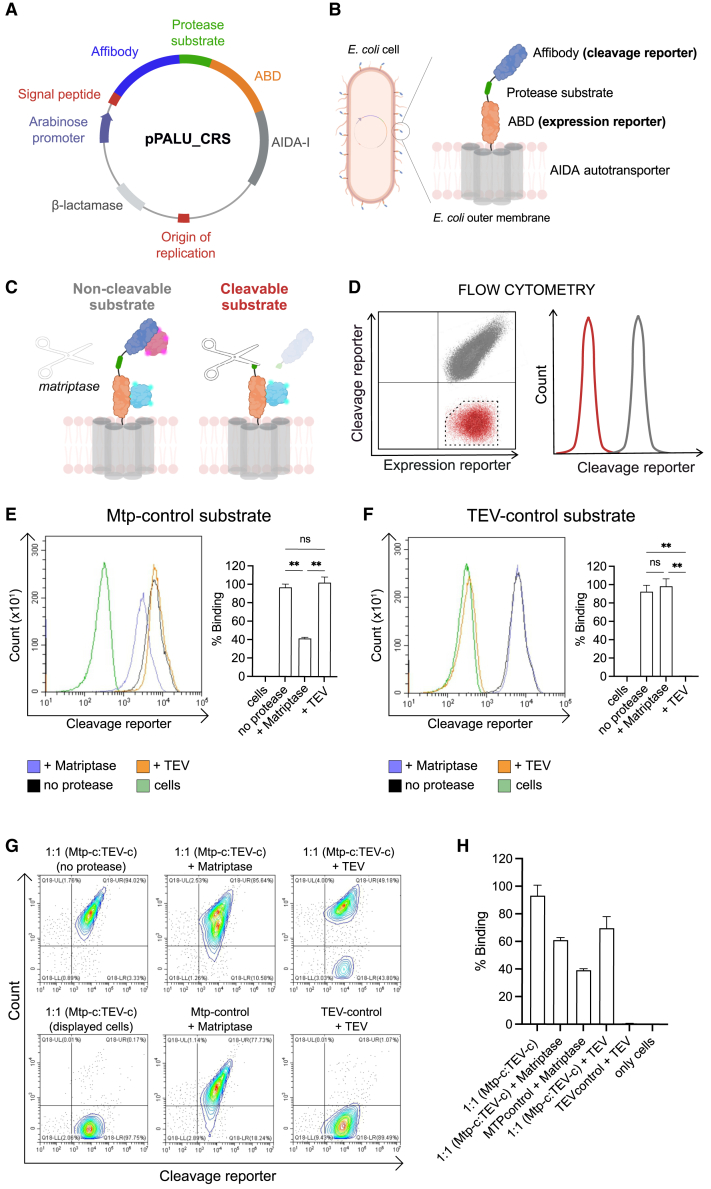
Design and analysis of the substrate cleavage reporter system using flow cytometry (A) Schematic of the plasmid (pPALU_CRS) encoding the cleavage reporter system. The plasmid includes an arabinose-inducible promoter for expression, an affibody domain (cleavage reporter), the protease substrate sequence, an albumin-binding domain (ABD) as the expression reporter, and a signal peptide for the outer membrane targeting using the AIDA-I autotransporter system. The plasmid also contains a β-lactamase gene for carbenicillin resistance and an origin of replication for plasmid propagation. (B) The expressed protein construct is displayed on the surface of *E. coli* cells via the AIDA-I autotransporter in a multivalent manner. The protein consists of an affibody domain (cleavage reporter), a protease substrate, and the ABD domain (expression reporter), all displayed on the cell surface. (C) Illustration of the cleavage assay principle. For cells displaying a non-cleavable substrate, the treatment with protease does not affect the cleavage reporter system, and signals from both the cleavage reporter (pink, HER2-biotin-SAPE) and the expression reporter (blue, HSA-Alexa647) can be detected by flow cytometry. For cells displaying a cleavable substrate, the cleavage reporter is removed after protease treatment, and only the signal from the expression reporter (blue) can be detected by flow cytometry. (D) Schematic representation of flow cytometric analysis of cells displaying cleavable (red) or non-cleavable (gray) substrates. A representative gate (black) is shown to indicate the population sorted by FACS. (E) Representative flow cytometry histograms of cells expressing the matriptase substrate (Mtp-control), treated with either matriptase or TEV protease. Cells stained with the cleavage reporter but not treated with protease served as a positive control (denoted: no protease), while cells treated with neither cleavage reporter (no HER2-biotin) nor protease served as a negative control (denoted: cells). The experiment was performed in triplicate (*n* = 3), and corresponding bar plots show the mean ± SD. Matriptase-treated cells exhibited significantly lower signals from cleavage reporter compared to both untreated cells (*p* = 0.005) and TEV-treated cells (*p* = 0.009). No significant difference was observed between TEV-treated and untreated cells (*p* = 0.38). (F) Representative flow cytometry histograms of cells expressing the TEV substrate (TEV-control), treated with either matriptase or TEV protease. Cells stained with the cleavage reporter but not treated with protease served as a positive control (denoted: no protease), while cells treated neither with cleavage reporter (no HER2-biotin) nor protease served as a negative control (denoted: cells). The experiment was performed in triplicate (*n* = 3), and corresponding bar plots show the mean ± SD. TEV-treated cells exhibited significantly lower signals from the cleavage reporter compared to both untreated cells (*p* = 0.009) and Mtp-treated cells (*p* = 0.009). No significant difference was observed between Mtp-treated and untreated cells (*p* = 0.47). (G) Flow cytometry contour plots showing cleavage reporter signals for a 1:1 mixture of cells displaying TEV-control and Mtp-control substrates treated with TEV or matriptase proteases. The corresponding controls were included for comparison. (H) The experiment was performed in triplicates (*n* = 3), and the bar plot indicates average and SD. Schematics were created with BioRender.com.

The main goal of this study was to identify substrate sequences for matriptase that could be cleaved more efficiently than previously reported substrates. To facilitate high-throughput screening of potential substrate sequences, we adapted a previously established method for displaying recombinant protein libraries on the surface of *E. coli*. We developed a display vector, named pPALU_CRS, using the pPALU vector^24^ as a template (Figure 1A). This new vector includes an autotransporter protein (AIDA-I), which anchors the displayed proteins to the bacterial outer membrane; a surface expression reporter domain (albumin-binding domain [ABD]^24^^,^^29^); and a cleavage reporter domain (HER2 binding affibody [Z_HER2_]^30^^,^^31^) separated by a flexible linker containing a protease substrate (Figures 1A and 1B). In this system, *E. coli* cells display the substrate on their surface and are thereafter exposed to a protease of interest (Figure 1C). When the substrate is cleaved by the protease, the cleavage reporter domain (Z_HER2_) dissociates from the cell surface. After protease treatment, the cells are labeled with fluorescent reagents targeting both the cleavage reporter domain (Z_HER2_; HER2-biotin-Streptavidin, R-Phycoerythrin conjugate, SAPE) and the expression reporter domain (ABD; HSA-Alexa647) (Figure 1C). The labeled cells are then analyzed via flow cytometry to quantify the extent of proteolytic cleavage. By combining this *E. coli* surface display with FACS, the method allows for high-throughput quantification of substrate cleavage efficiency, enabling discrimination and sorting of substrates based on their proteolytic susceptibility (Figures 1C and 1D). The method was first validated using matriptase and TEV protease, respectively.^32^ Substrates for each protease were subcloned into the new vector (pPALU_CRS) and expressed on *E. coli*. The different *E. coli* samples were treated with either tobacco etch virus (TEV) protease or matriptase, labeled with the detection reagents according to above, and analyzed by flow cytometry. Results confirmed that both substrates were successfully displayed on the cell surface, and each protease specifically cleaved its corresponding substrate, resulting in reduced fluorescence from the cleavage reporter domain (Figures 1E and 1F). Importantly, we observed simultaneous binding of both the cleavage and expression reporters (Figure 1G), indicating minimal steric hindrance between the labeled HSA and HER2, consistent with previous findings using the *E. coli* display system.^24^^,^^25^ No off-target cleavage was observed, i.e., TEV protease did not cleave the matriptase substrate, and matriptase did not cleave the TEV substrate (Figures 1E and 1F). Next, cells displaying the two substrates were mixed (1:1) and treated with TEV protease or matriptase followed by flow cytometric analysis. The results suggested that FACS could be effectively used to enrich substrate populations with diverse cleavage properties from combinatorial peptide libraries displayed on *E. coli* (Figures 1G and 1H).

### Design and characterization of a matriptase substrate library

The matriptase substrate library was designed based on previously reported amino acid preferences for matriptase substrates to bias the library toward sequences likely to be efficiently recognized and cleaved by matriptase (Figure S1).^15^^,^^33^^,^^34^ In particular, positively charged amino acids, such as arginine and lysine, were prioritized at the P1 position. Non-negatively charged residues were favored at the P1′ position, while small amino acids were prioritized at P2′ and hydrophobic residues at P3′. Cysteine residues were excluded from all positions to prevent the formation of disulfide bonds, which could interfere with substrate integrity. As prior studies have shown that the P4 and P4′ positions appear to have a minimal influence on matriptase cleavage, these were fixed to serine (P4) and glycine (P4′) to reduce overall library diversity. The resulting library had a theoretical diversity of 4.7 × 10^6^ variants, which was subcloned into the newly developed pPALU_CRS vector. The substrate library was transformed into *E. coli*, yielding 1.3 × 10^7^ transformants. Deep sequencing was employed to assess the sequence variability of the substrate library, and the observed amino acid frequencies at each substrate position (P3, P2, P1, P1′, P2′, and P3′) were found to be in close agreement with the theoretical design, as illustrated in Figure S1.

### FACS allows the enrichment of matriptase-cleavable substrates from a large library

**Figure 2:**
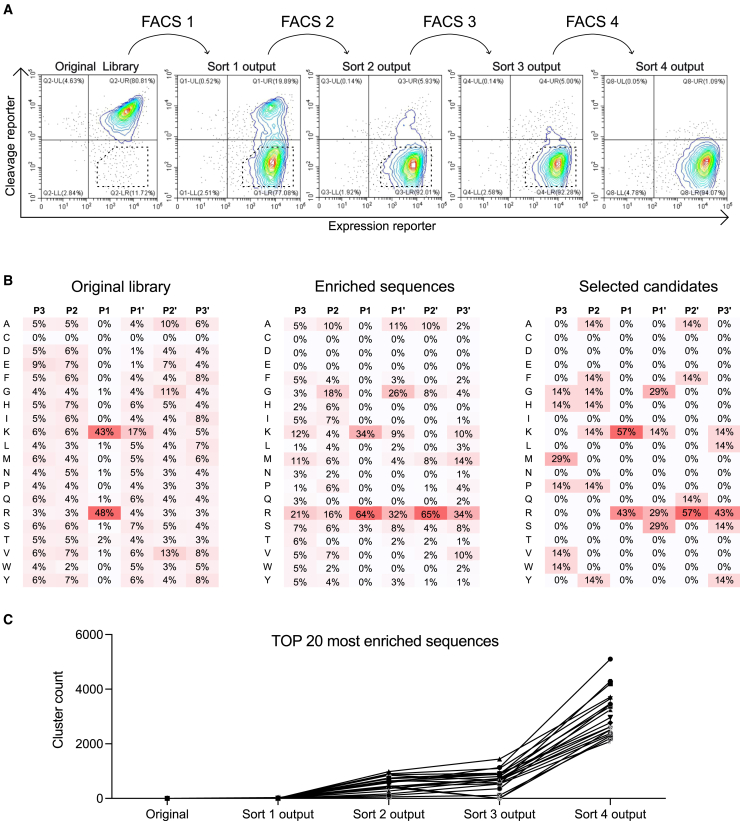
Flow cytometry analysis of library sorting outputs and sequencing results (A) Contour plots represent the flow cytometry analysis of the original library and fluorescence-activated cell-sorted outputs (sorts 1–4) treated with matriptase and labeled with both cleavage and expression reporters. The *x* axis corresponds to the signal from the expression reporter (ABD domain), indicating cell surface display levels, while the *y* axis corresponds to the signal from the cleavage reporter (affibody domain), representing whether the substrate has been cleaved. The sorting was performed by gating the cells located in the lower right quadrant. A representative gate is shown in the plots. (B) Amino acid frequencies at the substrate positions across the original library, sequences with >1,000-fold enrichment throughout the selection process, and selected candidates. The positions of the substrate sequence are denoted P3, P2, P1, P1′, P2′, and P3′, corresponding to the substrate residues surrounding the protease cleavage site. The frequency of each amino acid at these positions was determined by deep sequencing. The left image displays the amino acid distribution of the original library, the middle image illustrates the distribution of the most enriched sequences, and the right image presents the distribution of the selected candidates. The heatmap is color coded, with red indicating high amino acid frequency at a given position and white indicating low frequency. (C) Enrichment of the top 20 clusters sequences across sorting rounds. Cluster counts were determined from sequencing data of the original library and outputs from each of the four sorting rounds. Strong enrichment is observed after the final sort (sort 4 output), performed at increased stringency (10 nM matriptase).

The *E. coli*-displayed substrate library was treated with either matriptase or PBS (as a control) before being labeled with fluorescent reporters, as described above. Flow cytometry analysis revealed that, as expected, the majority of variants in the original library were not cleaved by matriptase (Figure 2A). To enrich for matriptase-cleavable variants, the library was subjected to four cycles of FACS. During successive FACS cycles, the concentration of matriptase was reduced (from 50 to 10 nM) to increase the stringency of the selection process. Flow cytometry analysis of the library outputs after each sorting cycle treated with matriptase showed progressively greater loss of the cleavage reporter, indicating enrichment of cleavable substrates (Figure 2A). By the final sorting cycle, the majority of the library consisted of linkers cleavable by matriptase. Negative controls, where the library was treated with PBS, showed no detectable cleavage (Figure S2).

### Deep sequencing analysis of sorted libraries reveals amino acid preferences in matriptase substrates

The original and sorted libraries were analyzed by deep sequencing. Library sizes and sequence cluster details are described in Table S1. The libraries were compared to evaluate the most enriched clusters (each consisting of sequences with 100% identity). The amino acid distribution at each position of the substrate (P3, P2, P1, P1′, P2′, and P3′) is shown in Figure 2B, with percentages indicating the frequency of each amino acid in these positions. In the original library, positions P3 and P2 were fully randomized. After sorting, a trend emerged, showing enrichment for amino acids with longer side chains at P3 (methionine [M], lysine [K], and arginine [R]) and small side chains at P2 (alanine [A] and glycine [G]). Negatively charged amino acids were largely disfavored across these positions. At position P1, both arginine (R) and lysine (K) were retained, but there was a preference for arginine over lysine. Additionally, positively charged amino acids, especially arginine, were enriched in other positions, suggesting that the cleavage site may have shifted in some substrates. In position P1′, a similar preference for arginine over lysine was observed, as well as a significant presence of glycine. Although P2′ was designed with a preference for small amino acids, this tendency was less obvious in the enriched clusters. The data indicated a general preference for hydrophobic (P3′) amino acids, in line with the design of the original substrate library. The sequencing data revealed strong enrichment of the top sequences following the final round of sorting, in which stringency was increased by reducing the matriptase concentration to 10 nM (Figure 2C).

### The display system is used to evaluate the cleavage efficiency of individual substrate candidates

**Figure 3:**
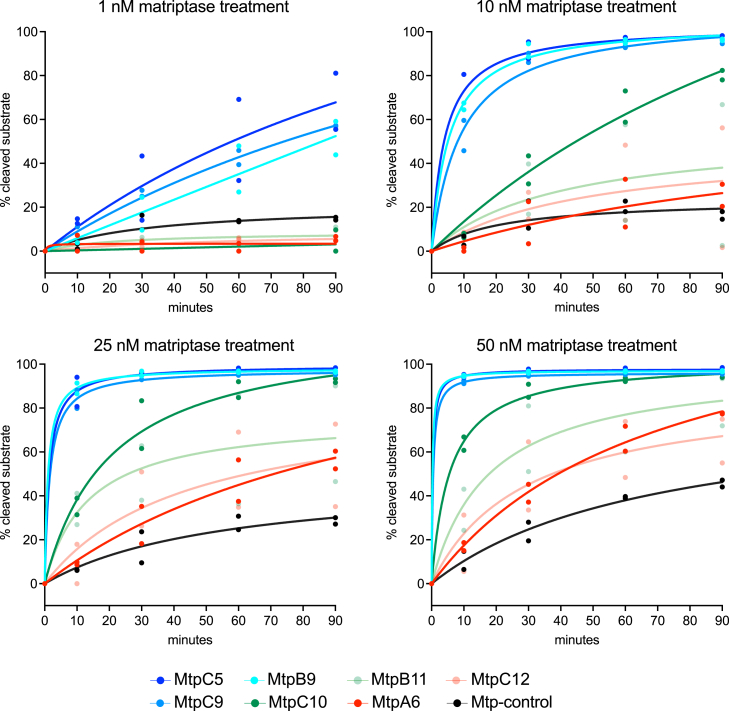
Cleavage kinetics of individual candidate substrates analyzed by flow cytometry using a cell surface display system The cleavage rate of each substrate candidate was measured at 0, 10, 30, 60, and 90 min following treatment with matriptase at varying concentrations (1, 10, 25, and 50 nM). Each graph represents the matriptase cleavage of different candidate substrates, with the fluorescence signal corresponding to the percentage of cleaved substrate at each time point. Substrate cleavage was monitored by flow cytometry, and data points are represented by two replicates.

From the sorted library, 192 randomly picked *E. coli* displaying candidate substrates were analyzed individually by flow cytometry to evaluate their substrate cleavage. After evaluation, seven candidates were selected for further study based on their cleavage profiles. Their cleavage rates were analyzed in detail at varying concentrations of matriptase (1, 10, 25, and 50 nM) over a period of 90 min (Figure 3). A previously described matriptase substrate (Mtp-control^5^) was included for comparison. The results showed that, even at a low matriptase concentration of 1 nM, over 50% of the best candidates (MtpB9, MtpC5, and MtpC9) were cleaved after 90 min of treatment. As expected, the cleavage rate increased with higher concentrations of matriptase, with MtpB9, MtpC5, and MtpC9 exhibiting the fastest cleavage. These candidates achieved complete cleavage in 60 min at 10 nM matriptase or within 30 min at 25 nM (Figure 3). Based on their relative cleavage efficiency, the candidates were categorized into three groups: high (MtpB9, MtpC5, and MtpC9), medium (MtpB11 and MtpC10), and low (MtpC12 and MtpA6). Notably, the low group displayed cleavage efficiencies comparable to the Mtp-control substrate.^5^ The amino acid sequence of the selected substrates is shown in Table S2.

### Cleavage analysis of soluble substrates in affibody-ABD format

*In vitro* cleavage was first evaluated by incorporating six of the previously selected substrates and controls into the linker region of a soluble affibody-ABD protein. The proteins were treated with matriptase, and the cleavage was analyzed by SDS-PAGE (Figures S3A and S3B). The results clearly demonstrated the superior cleavage efficiency of the newly identified substrates compared to both the matriptase control substrate and a non-cleavable substrate (TEV-control; Figures S3C and S3D). The new substrates exhibited partial cleavage (>50%) after just 10 min of treatment with 1 nM matriptase. Among these, MtpC5 had the highest cleavage efficiency, with approximately 86% cleavage within 10 min at 1 nM matriptase. In contrast, the Mtp-control substrate required 1 h of treatment at the highest concentration of matriptase (50 nM) to achieve 96% cleavage. As expected, the non-cleavable substrate (TEV-control) showed no cleavage under any tested conditions, further confirming the specificity of the cleavage reactions.

### FRET-based kinetic analysis of selected matriptase substrates reveals higher cleavage efficiency

**Figure 4:**
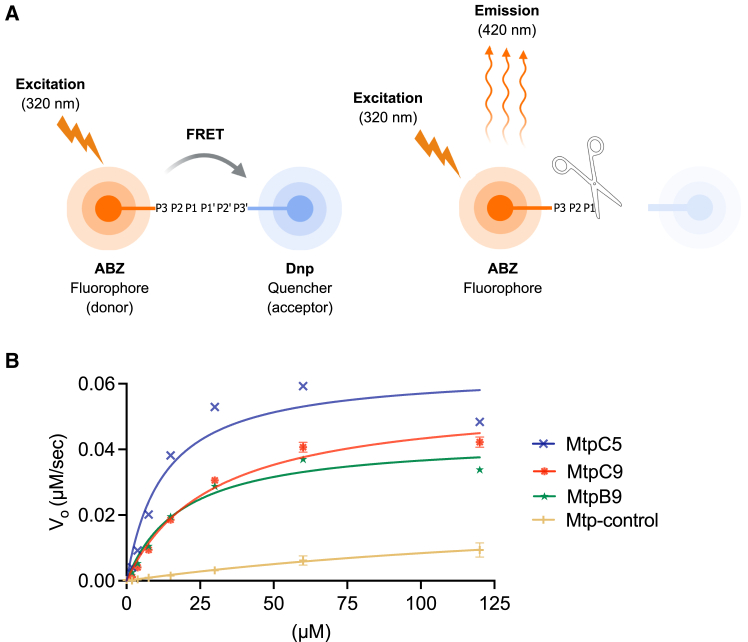
Schematic of FRET-based substrate cleavage detection and Michaelis-Menten kinetics of matriptase cleavage activity (A) Schematic representation of the FRET assay used to detect substrate cleavage by matriptase. The substrate consists of an Abz (fluorophore) at the N terminus and a Dnp (quencher) at the C terminus, separated by the substrate sequence (P3-P3′). In the intact substrate, FRET occurs between the fluorophore and quencher, reducing fluorescence emission. Upon cleavage of the substrate by matriptase at the P1-P1′ position, the quencher is separated from the fluorophore, resulting in an increase in fluorescence emission (420 nm) upon excitation at 320 nm. Schematics were created with BioRender.com. (B) Michaelis-Menten curves showing the relationship between substrate concentration and initial cleavage velocity (V_0_) for different substrate variants cleaved by matriptase. The curves illustrate the enzyme kinetics for each substrate variant. Data points represent the mean initial velocity (V_0_) at varying substrate concentrations, with fitted curves demonstrating the differences in cleavage efficiency across the substrates. Data points represent the mean ± standard deviation from three replicates. Error bars smaller than the size of the symbol are not shown.

The most promising newly discovered substrates (MtpB9, MtpC5, and MtpC9), along with control substrates, were chemically synthesized and linked to 2-aminobenzoyl (Abz) and 2,4-dinitrophenyl (Dnp), a standard fluorophore/quencher combination used in FRET assays (Figure 4A). A serial dilution of the substrates (ranging from 120 to 1.75 μM) was used to monitor matriptase cleavage activity. The reaction was tracked by measuring the Abz fluorescence continuously for 90 min. Kinetic parameters were calculated by fitting the cleavage rates to substrate concentrations using the Michaelis-Menten equation (Figure 4B). The kinetic results are summarized in Table 1 and correlate with the previous flow cytometry data, confirming that MtpC5 was the most efficiently cleaved substrate. The k_cat_/*K*_M_ value for MtpC5 (5.13E+05 ± 3,910 M^−1^ s^−1^) was 40-fold higher than that of the previously reported Mtp-control substrate^5^ (1.28E+04 ± 2,969 M^−1^ s^−1^). The result for the Mtp-control substrate was in good agreement with previously reported values, with 1.28E+04 M^−1^ s^−1^ in our measurements compared to 0.22E+04 M^−1^ s^−1^ in the previous study.^5^ The other candidates had k_cat_/*K*_M_ values showing 15- to 18-fold improvements over the control substrate.^5^

**Table 1:** Kinetic parameters of the selected matriptase substrates and Mtp-control

	k_cat_ (s^−1^)	*K*_M_ (μM)	V_max_ (μM/s)	k_cat_/*K*_M_ (M^−1^ s^−1^)	k_cat_/*K*_M_ fold change
MtpC5	6.4 ± 0.05	12.5	0.06 ± 0.0005	5.1E+05 ± 3,910	40.0
MtpC9	5.6 ± 0.21	29.1	0.06 ± 0.0021	1.9E+05 ± 7,099	15.0
MtpB9	4.4 ± 0.07	18.7	0.04 ± 0.0007	2.3E+05 ± 3,948	18.1
Mtp-control	2.5 ± 0.57	191.4	0.02 ± 0.0057	1.3E+04 ± 2,969	1.0

Average and standard deviation of experimental replicates (*n* = 3).

### Assessment of substrate stability against non-matriptase proteases

**Figure 5:**
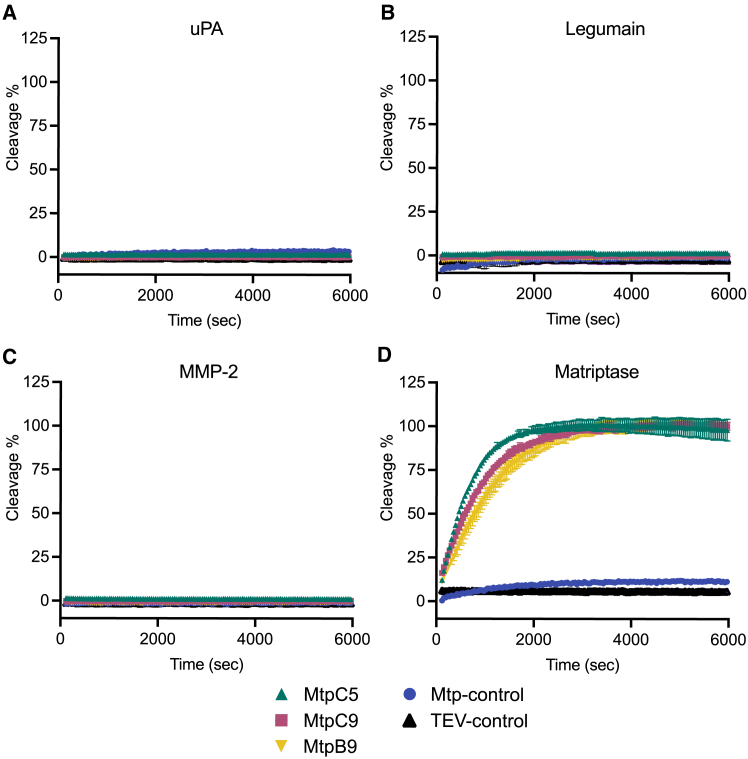
FRET-based measurement of substrate cleavage by different proteases confirmed substrate stability (A–C) Substrates were incubated with various proteases to assess their stability and specificity. Cleavage rates were monitored for 6,000 s in the presence of three non-matriptase proteases: (A) urokinase plasminogen activator (uPA), a serine protease; (B) legumain, a cysteine protease; and (C) MMP-2, a matrix metalloprotease. No detectable cleavage was observed with these proteases. (D) In contrast, significant cleavage was observed when the substrates were treated with matriptase. Substrate cleavage was monitored using FRET, and the percentage of cleaved substrate is plotted against the time for each protease. Substrates tested include MtpC5, MtpC9, MtpB9, Mtp-control, and TEV-control. Data points represent the mean ± standard deviation from three replicates.

The FRET substrate constructs (MtpB9, MtpC5, and MtpC9) were also used to assess substrate stability in the presence of various proteases. The substrates were incubated for 100 min (6,000 s) with legumain^35^ (cysteine protease), urokinase plasminogen^36^ (uPA; serine protease), and MMP-2^37^ (matrix metalloprotease), respectively. No detectable substrate cleavage was observed with any of these proteases (Figures 5A–5C). In contrast, cleavage was observed when the substrates were incubated with matriptase under identical conditions (Figure 5D).

### Prodrug activation was confirmed using matriptase-cleavable substrates

**Figure 6:**
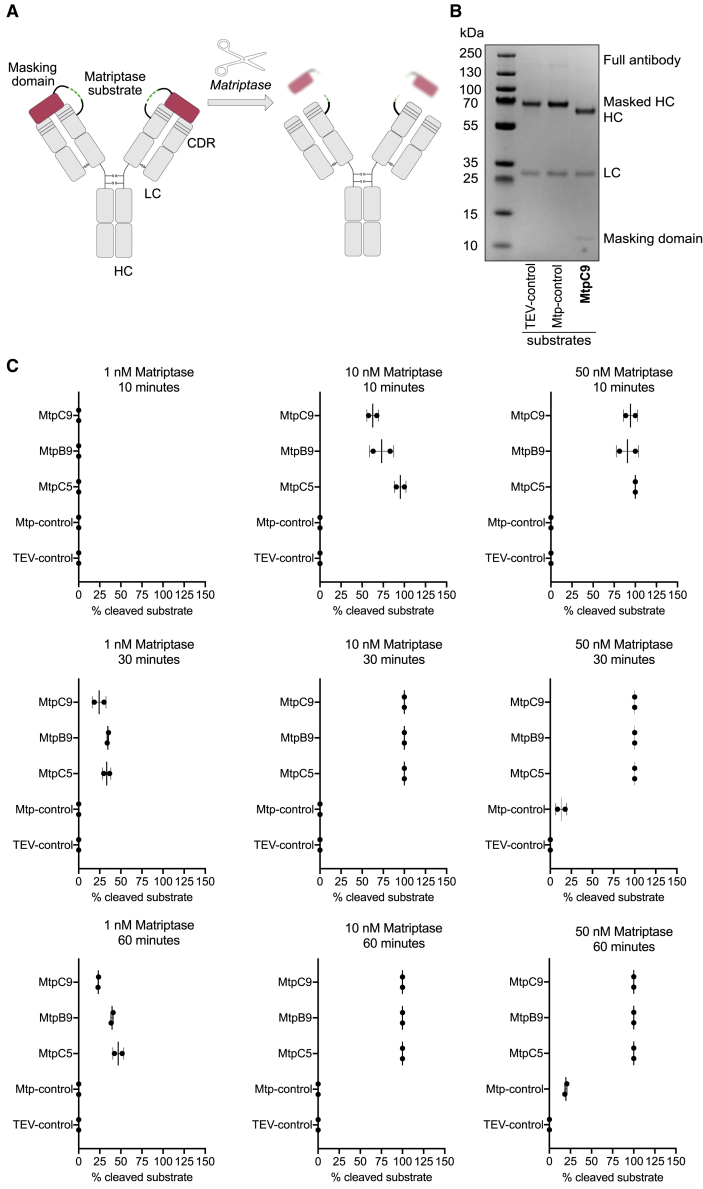
Evaluation of antibody prodrug cleavage by matriptase (A) Schematic representation of the antibody prodrug design, which includes a matriptase-cleavable substrate in the linker region between the antibody scaffold and the masking domain. Upon cleavage by matriptase, the masking domain is removed, activating the antibody. Schematics were created with BioRender.com. (B) SDS-PAGE analysis of antibody prodrugs after 30 min treatment with 10 nM matriptase. Bands corresponding to different components of the antibody are observed, including the full antibody prodrug (∼165 kDa), the masked heavy chain with the substrate and masking domain (∼57.5 kDa, Masked-HC), the cleaved heavy chain (∼50 kDa, HC), the light chain (∼25 kDa, LC), and the cleaved masking domain with the substrate (∼7.5 kDa, Masking domain). Samples include various substrates (MtpC9 shown as an example) and control groups (Mtp-control and TEV-control). (C) Quantitative analysis of substrate cleavage using three matriptase concentrations (1, 10, and 50 nM) over different incubation times (10, 30, and 60 min). Cleavage percentages for each substrate were calculated based on densitometric analysis of the SDS-PAGE results. Data points represent the mean ± standard deviation from two replicates.

Finally, the three best-performing substrates (MtpB9, MtpC5, and MtpC9) were incorporated into antibody prodrugs and evaluated for their ability to facilitate prodrug activation by matriptase (Figure 6A). The prodrugs consisted of an antibody fused to a masking domain via a linker containing the matriptase substrate. The Mtp-control substrate^5^ and a non-cleavable (TEV-control) substrate were included as positive and negative controls, respectively. The antibody prodrugs containing the various substrates were treated with matriptase at concentrations of 1, 10, and 50 nM for 10, 30, and 60 min, and the resulting cleavage was analyzed by SDS-PAGE (Figures 6B and 6C). SDS-PAGE revealed bands corresponding to the full antibody prodrug (∼165 kDa), the intact antibody heavy chain with substrate and masking affibody domain (∼57.5 kDa), the cleaved antibody heavy chain (∼50 kDa), the antibody light chain (∼25 kDa), and the cleaved masking affibody domain with substrate (∼7.5 kDa). The relative amount of cleaved substrate was quantified using ImageJ by analyzing the band intensity (Figure 6C). The results showed that the new substrates exhibited significantly more efficient cleavage than the Mtp-control in this prodrug format. Specifically, treatment with 1 nM matriptase resulted in partial cleavage (24.3%–34.6%) of the new substrates after just 30 min, whereas the Mtp-control showed no cleavage under these conditions (Figure 6C). Complete cleavage of MtpC5, MtpC9, and MtpB9 was achieved within 30 min at 10 nM matriptase. The control substrate required 50 nM matriptase and 60 min to achieve 19.5% cleavage. Overall, the results demonstrated that short treatment times and low matriptase concentrations (1 nM) were sufficient to activate prodrugs containing the new substrates. In all cases, 10 nM matriptase was sufficient to fully cleave the new substrates within 30 min. In contrast, the Mtp control showed no observable cleavage under the same conditions.

## Discussion

The primary aim of this study was to engineer matriptase substrates with enhanced cleavage efficiency to facilitate the development of next-generation biological prodrugs. To achieve this, we adapted an *E. coli* bacterial display system for high-throughput screening and characterization of protease substrates. This system utilized a substrate linker connecting a cleavage reporter domain and an expression reporter domain on the bacterial outer membrane, allowing quantitative assessment of protease activity through flow-cytometric analysis. Initial validation demonstrated that the platform could accurately differentiate between substrates based on cleavage efficiency, establishing a robust tool for high-throughput substrate evaluation. Although the *E. coli* display is less commonly used than, for example, phage or ribosome display, it offers certain advantages. Its multivalent presentation of substrates, combined with the ability to sort single cells by FACS, enables high-throughput selection of protease substrates based on quantitative cleavage data, as well as assessment of their relative cleavage rates. In contrast, phage and ribosome display platforms are typically monovalent, which limits their capacity for quantifying substrate cleavage and restricts analysis to a more qualitative readout.

Following initial validation, we generated a library of over 4 million substrate variants. As the primary goal was to identify improved matriptase substrates, the library was designed based on known substrate preferences, with partial randomization from positions P3′ to P3 while keeping P4 and P4′ fixed to limit overall diversity. While this biased approach increases the likelihood of discovering high-efficiency substrates, a fully randomized library would be a compelling alternative for future studies aimed at characterizing less-explored proteases or uncovering novel substrate specificities. Similarly, performing selections and screenings under alternative conditions, such as lower pH, could provide insights into how environmental factors influence the protease’s substrate preference.

The designed library was displayed on *E. coli* cells in order to select candidates that could be cleaved by matriptase. After four rounds of FACS, the library was significantly enriched for cleavable substrates. Deep sequencing of both the original and sorted libraries confirmed that sequence variability in the initial library aligned well with the design while also revealing amino acid preferences for matriptase cleavage (Figure 2). Over 190 randomly picked hits were ranked for cleavage efficiency using the high-throughput flow cytometry assay, and seven candidates were selected for detailed cleavage analysis (Figure 3). The best-performing substrates were chemically synthesized and conjugated to a fluorophore-quencher pair for kinetic evaluation, with a previously reported matriptase substrate^5^ included for comparison (Figure 4). Kinetic parameters were determined (Table 1), and k_cat_/*K*_M_ values were calculated for each substrate, ranging from 1.92E+05 to 5.13E+05 M^−1^s^−1^, with all new substrates demonstrating substantially higher values compared to the reference substrate Mtp-control. Notably, the most efficient substrate, MtpC5, exhibited a k_cat_/*K*_M_ value 40 times higher than the reference substrate, indicating significantly enhanced cleavage efficiency. Interestingly, the P3-to-P3′ sequence of the control substrate (SGRSDN) was not recovered in any of the sorted outputs, probably due to its lower cleavage efficiency relative to the newly identified substrates. Moreover, the substrates used in the FRET assay were strictly six amino acids long, with no flanking peptide linkers, demonstrating that matriptase efficiently cleaves short 6-mer substrates. The strong correlation between results from the *E. coli* display system and subsequent biochemical assays confirmed the reliability of the high-throughput method for both protease substrate discovery and cleavage efficiency evaluation. However, each characterization method offers distinct advantages and limitations. The FRET assay is well suited for determining enzyme kinetics and substrate cleavage efficiency but requires chemically synthesized substrates, which is both time consuming and costly. SDS-PAGE is a straightforward technique; however, its quantification is limited and typically relies on imaging software for relative band intensity analysis. The *E. coli* display combined with flow cytometry is highly effective for parallel validation of cleavage across many substrate variants. It allows for straightforward evaluation at different protease concentrations and time points. However, the lack of control over substrate concentration limits its utility for deriving kinetic parameters, restricting the method to relative quantification and ranking. Overall, we consider the combination of the high-throughput *E. coli* display and FRET analysis a powerful strategy, using the *E. coli* display to screen and prioritize candidates, followed by detailed kinetic characterization of the top hits using synthetic FRET substrates.

The newly identified substrates were also tested against other proteases as an initial assessment of their general stability. However, substrate stability is ultimately determined by the protease environment relevant to the intended application. Consequently, depending on how these substrates will be used, their stability and selectivity should be thoroughly evaluated under appropriate physiological or experimental conditions.

Finally, the selected substrates were incorporated into an antibody prodrug format to evaluate their ability to trigger prodrug activation *in vitro*. The results demonstrated that even at low matriptase concentrations (1 nM), the new substrates were efficiently cleaved, leading to the removal of the masking domain and subsequent prodrug activation. In conclusion, this study successfully validated the use of an *E. coli* display platform for high-throughput protease substrate engineering. The platform proved to be a powerful tool for screening and optimizing protease substrates, and it holds promise for discovering new ones. The matriptase substrates identified in this study exhibited k_cat_/*K*_M_ values over 40-fold higher than the best previously reported substrates, highlighting their potential for use in future prodrug applications. Importantly, the diverse set of identified substrates, with varying cleavage efficiencies, provides opportunities to tailor cleavage profiles to suit specific requirements of different end-use applications. These findings provide a solid foundation for the further development of protease-activated prodrugs, with the potential to improve therapeutic efficacy and tissue selectivity in cancer therapies and other targeted treatments. Additionally, the identified substrates could also be adapted for diagnostic applications to quantify and detect active matriptase *in vivo* or *ex vivo*. This would be highly valuable, as measuring active protease levels in tissues remains a significant challenge and could offer important insights for the development of new therapeutic strategies. Moreover, the platform may facilitate the discovery of protease inhibitors, which is an established therapeutic class already in use for treating a range of diseases, including cancer, neurodegenerative disorders, and infections.

### Limitations of the study

The bacterial display system enables high-throughput identification of protease substrates, but it has some limitations. The use of *E. coli* may restrict the display of certain sequences due to folding or expression constraints. The screening is performed under *in vitro* conditions that may not fully mimic the tumor microenvironment. While the method allows relative ranking of cleavage efficiency, detailed kinetic characterization still requires follow-up assays. Moreover, the library design was biased toward known matriptase preferences, potentially limiting the discovery of novel substrate motifs.

## Resource availability

### Lead contact

Further information and requests for resources and reagents should be directed to and will be fulfilled by the lead contact, John Löfblom (lofblom@kth.se).

### Materials availability

The amino acid sequences for the new protease substrate sequences are available in Table S2.

### Data and code availability

• Next-generation sequencing data have been deposited in the Mendeley Data repository under https://doi.org/10.17632/tm79cffy8m.1. All other data reported in this paper will be shared by the lead contact upon request.
• This paper does not report original code.
• Any additional information required to reanalyze the data reported in this paper is available from the lead contact upon request.

## Acknowledgments

This work was supported primarily by grants from the 10.13039/501100002794Swedish Cancer Society (CAN 23 2717 Pj, JL 20 1090 PjF, and JL 19 0101 Pj01H) and the 10.13039/501100004063Knut and Alice Wallenberg Foundation (grants KAW 2019.0341, KAW 2021.0197, KAW 2023.0073, and KAW 2023.0512) and Wallenberg Center for Protein Research (WCPR). Additional support was provided by the Swedish Cancer Society (SS 22 2023 Pj01H, S.S.), 10.13039/501100003792Hjärnfonden (FO2022-0253 and FO2023-0141, J.L.), the 10.13039/501100004359Swedish Research Council (2019–05115, J.L.), StratNeuro (J.L.), and the Swedish Agency for Innovation VINNOVA (2019/00104 and CellNova Center: 2017/02105, J.L.). The authors acknowledge support from the National Genomics Infrastructure in Stockholm funded by the 10.13039/501100009252Science for Life Laboratory, the Knut and Alice Wallenberg Foundation, and the Swedish Research Council, and we acknowledge SNIC/Uppsala Multidisciplinary Center for Advanced Computational Science for assistance with massively parallel sequencing and access to the UPPMAX computational infrastructure. The authors also thank Elin V.W. for her contributions to the preliminary experiments of this project.

## Author contributions

Conceptualization, A.M.B., S.S., and J.L.; investigation, A.M.B. and H.M.; formal analysis, A.M.B. and H.M.; visualization, A.M.B. and J.L.; writing – original draft, A.M.B.; writing – review & editing, A.M.B., H.M., S.S., and J.L.; funding acquisition, S.S. and J.L.; supervision, J.L.

## Declaration of interests

A.M.B., J.L., and S.S. are shareholders of Zytox Therapeutics AB. J.L. and S.S. are board members of Zytox Therapeutics AB. A.M.B. and J.L. have a patent application related to this work owned by Zytox Therapeutics AB.

## STAR★Methods

### Key resources table

**Table:** 

REAGENT or RESOURCE	SOURCE	IDENTIFIER
**Bacterial and virus strains**		

*Escherichia coli* BL21 (DE3)	New England Biolabs	C2527I

**Chemicals, peptides, and recombinant proteins**		

HER2/ERBB2 Protein ECD, His Tag	SinoBiologicals	10004-H02H
Human serum albumin	Kabivitrum	
Alexa Fluor™ 647 NHS Ester	Invitrogen	A20006
Matriptase	R&D systems/Bio-Techne	3946-SEB
Urokinase-type plasminogen activator	R&D systems/Bio-Techne	1310-SE
Legumain	R&D systems/Bio-Techne	2199-CY
Matrix metalloproteinase-2	R&D systems/Bio-Techne	902-MP
Tobacco etch virus protease	In-house produced	N/A
4-Aminophenylmercuric acetate	Sigma-Aldrich	164610
NcoI	New England Biolabs	R0193S
SpeI	New England Biolabs	R3133S
Streptavidin R-Phycoerythrin Conjugate (SAPE)	Thermo Fisher Scientific	S866
Phusion polymerase	Thermo Fisher Scientific	F530S
FRET substrate with 2-Aminobenzoyl (Abz) 2,4-dinitrophenyl (Dnp)	Bachem	Custom order

**Critical commercial assays**		

Biotin-XX Microscale Protein Labeling Kit	Thermo Fisher Scientific	B30010
Qiagen Miniprep Kit	Qiagen	27104
Qiagen Gel Extraction Kit	Qiagen	28704
Qubit™ dsDNA High Sensitivity and Broad Range Assay kit	Invitrogen	Cat#Q33266
In-Fusion® HD Cloning Kit	Takara	102518
PD-10 desalting columns	Cytiva	17085101
ExpiFectamine™ CHO Transfection Kit	Thermo Fisher Scientific	A14524
HiTrap PrismA column	Cytiva	17549854

**Deposited data**		

NGS data of original library and sort outputs 1-4	Mendeley Data repository	https://doi.org/10.17632/tm79cffy8m.1

**Experimental models: Cell lines**		

Hamster: ExpiCHO-S cells	Thermo Fisher Scientific	A29127

**Oligonucleotides**		

Primer P1 (forward primer): AATGATACGGCGACC ACCGAGATCTACACTCTTTCCCTACACGACGCTC TTCCGATCTTTAAACAATCAGCAAAAGCGCG	Thermo Fisher Scientific	Custom order
Primer P2 (reverse primer), TruSeq index in bold (example Index 1): CAAGCAGAAGACGGCATACGAG AT**CGTGAT**GTGACTGGAGTTCAGACGTGTGCTCTT CCGATCGAAGAACTTTTGCTTCTGCCAGACT	Thermo Fisher Scientific	Custom order

**Recombinant DNA**		

pET26b vector	Novagen-Merck	69862
pPALU_CRS	This study	Modified from pPALU in Parks et al.^24^
DNA encoding G_4_SG_4_S-P3 P2 P1 P1′ P2′ P3′- G_4_SG_4_S	Ella Biotech GmbH	Custom order
pcDNA3.1-derived vector	Thermo Fisher Scientific	V79020

**Software and algorithms**		

PipeBio	Benchling	N/A
Python version 3.9.12	N/A	www.python.org
Microsoft Excel version 16.86	Microsoft	N/A
GraphPad Prism 10	GraphPad	N/A
ImageJ version 1.54i	ImageJ	N/A
BioRender	Biorender	N/A

### Experimental model and study participant details

#### Bacterial strains

*Escherichia coli* BL21 (DE3) was used for all cloning and expression experiments involving TEV protease, substrate display, and affibody-ABD fusion constructs.

#### Cell lines

ExpiCHO-S cells (Thermo Fisher Scientific) were used for recombinant antibody prodrug expression.

### Method details

#### Recombinant proteins and protein labeling

Recombinant human epidermal growth factor receptor 2 (HER2/ERBB2 Protein ECD, His Tag, SinoBiologicals, China) was biotinylated using the Biotin-XX Microscale Protein Labeling Kit (Thermo Fisher Scientific, US) according to the manufacturer’s protocol. Human serum albumin (HSA, Albumina Kabi 20%, Kabivitrum, Sweden) was conjugated with the Alexa Fluor 647 dye following the manufacturer’s instructions (Alexa Fluor 647 NHS Ester, Invitrogen, US). Recombinant proteases, including matriptase, urokinase-type plasminogen activator (uPA), legumain, and matrix metalloproteinase-2 (MMP-2), were obtained from R&D Systems (Bio-Techne, US). Prior to use, human MMP-2 was activated by incubation with 0.2 mM 4-Aminophenylmercuric acetate (APMA) at 37°C for 1 h in MMP assay buffer (50 mM Tris, 10 mM CaCl_2_, 150 mM NaCl, 0.05% Triton X-1000, pH 7.5). Tobacco etch virus (TEV) protease was produced in-house by recombinant expression in Escherichia coli BL21 (DE3) (New England Biolabs, US). Briefly, the gene encoding TEV protease was fused to a hexahistidine-tag and cloned into a modified pET26b vector (Novagen-Merck, Germany). Expression was induced in E. coli, and the proteins were extracted via sonication (1.5 min, 1 s ON/1 s OFF cycles) from the cytoplasm. TEV protease was purified using a HiTrap TALON column (Cytiva, Sweden) in an ÄKTA Start system (Cytiva) in accordance with the manufacturer’s protocol.

#### Construction of a substrate cleavage reporter system

The genes encoding the substrate display system were cloned into a modified version of the pPALU vector, hereinafter referred to as pPALU_CRS (cleavage reporter system) (Figure 1A). In all cases, the substrates were flanked by a flexible linker sequence at each side (G4SG4S) to avoid steric hindrance from the reporter domains. Genes encoding for the TEV substrate (TEV-control; ENLYFQG) and a reference matriptase substrate (Mtp-control; LSGRSDNH) were subcloned to the vector by restriction enzyme cloning using NcoI and SpeI following manufacturer’s recommendations (New England Biolabs, US). Each plasmid was individually transformed into *E. coli* BL21 (DE3) cells using a standard heat-shock protocol. After transformation, the cells were plated on agar supplemented with carbenicillin (100 μg/mL), and single colonies were selected to inoculate 5 mL of Luria-Bertani (LB) media supplemented with carbenicillin (100 μg/mL). The cultures were grown overnight at 37°C, 150 rpm. Glycerol stocks of each variant (20% glycerol solution) were prepared and stored at −80°C. Subsequently, DNA encoding the matriptase library (G4SG4S-P3 P2 P1 P1′ P2′ P3′-G4SG4S) was synthesized by Ella Biotech (Germany) and subcloned by NcoI and SpeI into the pre-digested pPALU_CRS vector. The library was transformed into *E. coli* BL21 (DE3) cells via electroporation. The library size was estimated by plating transformation dilutions on carbenicillin agar after 1-h incubation. Transformed cells were grown in LB media supplemented with carbenicillin (100 μg/mL) overnight at 37°C, 150 rpm. Glycerol stocks of the library were prepared and stored at −80°C. Library diversity was assessed by deep sequencing using the TruSeq MiSeq Illumina platform (Illumina, US) through the National Genomics Infrastructure (NGI, Sweden).

#### Library labeling and sorting by flow cytometry

A volume of the glycerol stock sufficient to encompass the library’s diversity at least 10x was inoculated into fresh LB media supplemented with carbenicillin (100 μg/mL). The cultures were grown overnight, diluted to an OD600 of 0.1 in fresh LB media, and induced with 0.6% arabinose once they reached an OD600 of 0.7. After overnight incubation, the induced cell cultures were harvested, washed twice with PBS supplemented with 1% Pluronic acid (PBS-P), and treated with matriptase (50–10 nM) for 30 min at 37°C. Matriptase concentration was progressively reduced across selection rounds to increase stringency. After protease treatment, cells were washed twice with PBS-P and incubated with 50 nM biotinylated HER2 for 1 h at room temperature. Subsequently, the cells were incubated with 225 nM Alexa Fluor 647-HSA and 33.3 nM Streptavidin, R-Phycoerythrin conjugate (SAPE) in PBS-P for 30 min at 4°C. After incubation, cells were washed twice with PBS-P and either analyzed using a Cytoflex S flow cytometer (Beckman Coulter, US) or sorted using a Cytoflex SRT instrument. The sorting gate was placed to retain cells with expression reporter signal but reduced cleavage reporter signal. Sorted cells were grown in LB media with carbenicillin, aliquoted as glycerol stocks, and stored at −80°C. Single colonies from plated dilutions were picked for further analysis.

#### Deep sequencing analysis of sorting outputs

Overnight cultures from the sorted libraries were used for plasmid extraction using the Qiagen Miniprep Kit (Qiagen, US). The purified plasmids served as templates for PCR amplification of the substrate regions, and PCR was carried out using Phusion polymerase (Thermo Fisher Scientific, US). The forward primer (primer P1) contains a TruSeq-compatible adapter sequence followed by a universal binding region targeting a conserved upstream sequence in all constructs. The reverse primers (primer P2) each contained a construct-specific binding region and a unique TruSeq index adapter to enable multiplexing (Illumina, US). The PCR products were purified using the Qiagen Gel Extraction Kit (Qiagen, US), and their concentration was quantified using the Qubit fluorometer (Thermo Fisher Scientific, US). Sequence variability of the sorted libraries was analyzed via deep sequencing on the TruSeq MiSeq Illumina platform at the National Genomics Infrastructure (NGI, Sweden). Libraries were annotated to identify protease substrate regions and clustered based on 100% similarity. The enrichment of sequences across selection rounds was analyzed, and amino acid frequency at each substrate position was calculated for sequences with >1000-fold enrichment. Data were processed using PipeBio (Benchling, US), Python (v3.9.12), Microsoft Excel (v16.86), and GraphPad Prism 10 (GraphPad, US).

#### Flow cytometry analysis of displayed individual candidates

*E. coli* cells displaying Mtp-control and TEV-control substrates were grown overnight in LB media with carbenicillin (100 μg/mL). Cultures were diluted to OD600 = 0.1 and induced with 0.6% arabinose at OD600 = 0.7. After overnight induction, 10 μL of each culture was transferred to 96-well conical plates. For mixed population analysis, a 1:1 mix of cultures was used. Cells were washed twice with PBS-P and treated with 100 nM matriptase (30 min, 37°C) or 100 nM TEV (30 min, 30°C). Cells were then labeled with 50 nM biotinylated HER2 for 1 h at room temperature, followed by incubation with 225 nM Alexa Fluor 647-HSA and 33.3 nM SAPE in PBS-P (30 min, 4°C). Cells were analyzed using a Cytoflex S flow cytometer. Randomly selected clones from FACS outputs were also analyzed. For kinetics, clones were treated with 0–50 nM matriptase and sampled at 10, 30, 60, or 90 min. Cleavage (%) was determined by loss of cleavage reporter relative to untreated controls. Negative values were set to zero. Michaelis-Menten curves were fitted using Prism 10, and one-way ANOVA was used to compare cleavage profiles.

#### Production and purification of soluble substrates in affibody-ABD format

Substrate sequences were cloned into pET vectors encoding affibody-ABD fusion proteins. Controls included Mtp-control and TEV-control substrates. Cloning was performed with the In-Fusion HD Cloning Kit (Takara, Japan). Verified plasmids were transformed into *E. coli* BL21 (DE3). Expression was induced with 1 mM IPTG in TSB-Y medium with kanamycin (50 μg/mL) at OD600 = 0.7, followed by overnight incubation at 25°C. Cells were lysed by sonication, and lysates clarified by centrifugation (25,000 × g, 15 min, 4°C). Proteins were purified using HisPur Cobalt resin (Cytiva, Sweden) and desalted into PBS using PD-10 columns. SDS-PAGE (NuPAGE) was used to confirm purity, stained with GelCode Blue, and imaged with a GelDoc system (Bio-Rad, US).

#### Cleavage analysis of soluble substrates in affibody-ABD format

Affibody-ABD substrates (50 μg/mL in PBS) were treated with 1, 10, or 50 nM matriptase at 37°C for 10, 30, or 60 min. Reactions were quenched by adding SDS loading buffer (31.2 mM Tris-HCl, pH 6.8, 1% SDS, 12.5% glycerol, 0.01% bromophenol blue, 5 mM TCEP) and heating at 90°C for 10 min. Samples were analyzed by SDS-PAGE as above.

#### Characterization of substrate kinetics and specificity by FRET

Top substrates were synthesized with 2-aminobenzoyl (Abz) and 2,4-dinitrophenyl (Dnp) labels (Bachem, Germany). Peptides were dissolved in DMSO (15 mM) and stored at −80°C. For assays, substrates were diluted to 600 nM in PBS. Serial dilutions (0–120 nM) were incubated with 10 nM matriptase in 96-well black plates. Fluorescence (Ex: 320 ± 9 nm, Em: 420 ± 9 nm) was recorded over 90 min at 37°C using a Clariostar reader (BMG Labtech). Initial velocity (V0) was calculated from early linear slopes. Michaelis-Menten parameters (Vmax, Km, kcat) were determined using GraphPad Prism. Protease specificity was tested by incubating 30 μM peptide with 10 nM of each protease (matriptase, uPA, legumain, MMP-2) in appropriate buffer at 37°C. Fluorescence was recorded for 6000 s.

#### Production of substrates in antibody prodrugs

Candidate substrates were cloned into a pcDNA3.1-derived vector containing a masking domain linked to the cetuximab VH chain via a cleavable linker. Controls included TEV-control and Mtp-control. Constructs were expressed in ExpiCHO cells using ExpiFectamine CHO reagents (Thermo Fisher Scientific, US). After 12 days, supernatants were harvested by centrifugation (4000 × g, 30 min) and filtered (0.2 μm). Antibodies were purified using HiTrap PrismA columns (Cytiva, Sweden), buffer exchanged into PBS, and stored at −20°C.

#### Cleavage analysis of substrates in prodrugs

Antibody prodrugs (50 μg/mL) were treated with 1, 10, or 50 nM matriptase at 37°C for 10, 30, or 60 min. Reactions were quenched with SDS loading buffer + TCEP and analyzed by SDS-PAGE.

#### SDS-PAGE gel quantification by ImageJ

High-resolution gel images (600 dpi) were analyzed using ImageJ (v1.54i). Band intensities were quantified to assess the fraction of cleaved vs. intact substrate. Data were processed in Microsoft Excel and visualized with GraphPad Prism 10.

#### Other tools

Schematic figures were created using BioRender (BioRender.com). All data plots were generated using GraphPad Prism 10 (GraphPad, US).Relative cleavage was calculated based on densitometry, with raw values processed in Excel and plotted in GraphPad Prism.

### Quantification and statistical analysis

All flow cytometry and gel quantification data were analyzed using GraphPad Prism 10. Michaelis-Menten fits were used to derive kinetic parameters. One-way ANOVA was applied to compare cleavage efficiencies between different proteases. For all experiments, a minimum of three biological replicates were performed unless stated otherwise.

## Supplemental information

Document S1. Figures S1–S3 and Tables S1 and S2

Document S2. Article plus supplemental information
