# Supplementary material for: Engineering high-efficiency matriptase substrates using E. coli display for applications in prodrug activation
Source: Cell Rep Methods. 2025 Jun 10;5(6):101077. doi: 10.1016/j.crmeth.2025.101077 (PMC12272249; doi:10.1016/j.crmeth.2025.101077)
Supplement: Document S1. Figures S1–S3 and Tables S1 and S2 [file mmc1.pdf]

Cell Reports Methods, Volume *5*

Supplemental information

Engineering high-efficiency matriptase substrates using *E. coli* display for applications in prodrug activation

Anna Mestre Borras, Hanna Mehari, Stefan Ståhl, and John Löfblom

SUPPLEMENTAL MATERIAL

Supplementary Fig. 1. Heat map comparing amino acid frequencies in the theoretical (designed) and original (sequenced) substrate libraries. Related to STAR Methods. The heat maps display the amino acid frequencies at each substrate position for both the theoretical library (left) and the original sequenced library (right), as analyzed by deep sequencing. The positions of the substrate sequence are denoted as P3, P2, P1, P1', P2', and P3', corresponding to the substrate residues surrounding the protease cleavage site. Red shading indicates high amino acid frequency, while white represents low frequency. This comparison illustrates the differences between the designed diversity and the actual representation of amino acids in the experimental library.

Theoretical library

	P3	P2	P1	P1'	P2'	P3'
A	5%	5%	0%	5%	12%	7%
C	0%	0%	0%	0%	0%	0%
D	5%	5%	0%	1%	4%	3%
E	5%	5%	0%	1%	4%	3%
F	5%	5%	0%	5%	4%	8%
G	5%	5%	0%	5%	12%	4%
H	5%	5%	0%	5%	4%	4%
I	5%	5%	0%	5%	4%	7%
K	5%	5%	40%	18%	4%	4%
L	5%	5%	0%	5%	4%	7%
M	5%	5%	0%	5%	4%	7%
N	5%	5%	0%	5%	4%	4%
P	5%	5%	0%	5%	4%	4%
Q	5%	5%	0%	5%	4%	4%
R	5%	5%	60%	5%	4%	4%
S	5%	5%	0%	5%	4%	4%
T	5%	5%	0%	5%	4%	4%
V	5%	5%	0%	5%	12%	7%
W	5%	5%	0%	5%	4%	8%
Y	5%	5%	0%	5%	4%	7%

Original library

	P3	P2	P1	P1'	P2'	P3'
A	5%	5%	0%	4%	10%	6%
C	0%	0%	0%	0%	0%	0%
D	5%	6%	0%	1%	4%	4%
E	9%	7%	0%	1%	7%	4%
F	5%	6%	0%	4%	4%	8%
G	4%	4%	1%	4%	11%	4%
H	5%	7%	0%	6%	5%	4%
I	5%	6%	0%	4%	4%	8%
K	6%	6%	43%	17%	4%	5%
L	4%	3%	1%	5%	4%	7%
M	6%	4%	0%	5%	4%	6%
N	4%	5%	1%	5%	3%	4%
P	4%	4%	0%	4%	3%	3%
Q	6%	4%	1%	6%	4%	4%
R	3%	3%	48%	4%	3%	3%
S	6%	6%	1%	7%	5%	4%
T	5%	5%	2%	4%	3%	3%
V	6%	7%	1%	6%	13%	8%
W	4%	2%	0%	5%	3%	5%
Y	6%	7%	0%	6%	4%	8%

Supplementary Fig. 2. Flow cytometry analysis of the original library and first sorting cycle treated with either PBS or 50 nM Matriptase. Related to STAR Methods.

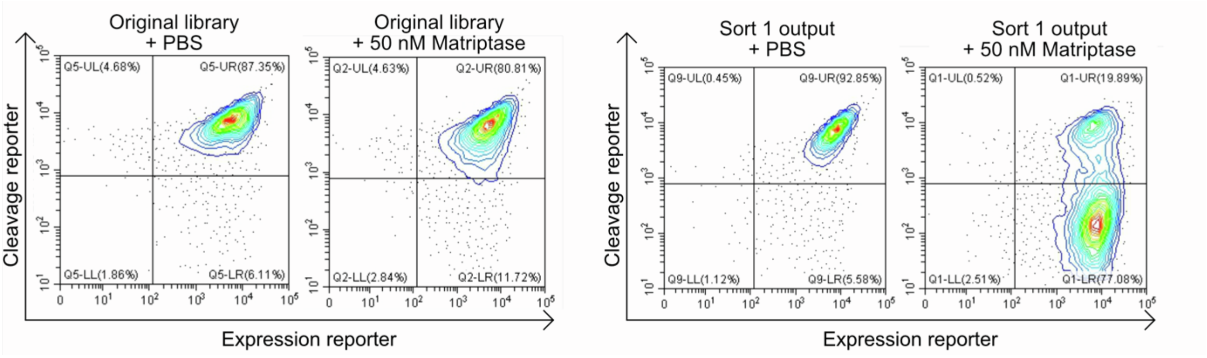

Supplementary Fig. 3. Cleavage analysis of soluble substrates in affibody-ABD format. Related to STAR Methods.

a Schematic representation of the affibody-ABD protein used as a soluble substrate for matriptase cleavage. **b** SDS-PAGE analysis of affibody-ABD cleavage exemplified with MtpC9, Mtp-control and TEV-control. The gel bands correspond to the full protein (above 16 kDa), cleaved affibody domain (~10 kDa), and cleaved ABD domain (~6 kDa). The samples were treated for 10 min with 10 nM matriptase**.** **c** Analysis of the cleavage reaction, quantified by measuring the relative band intensities using ImageJ software. Curves represent the percentage of cleaved substrate at different time points (0, 10, 30 and 60 min) and matriptase concentrations (1, 10 and 50 nM), illustrating the cleavage efficiency of the affibody-ABD protein. Data points are represented by two replicates. **d** Cleavage rates from (c) shown individually. Data points represent the mean ± standard deviation from two replicates.

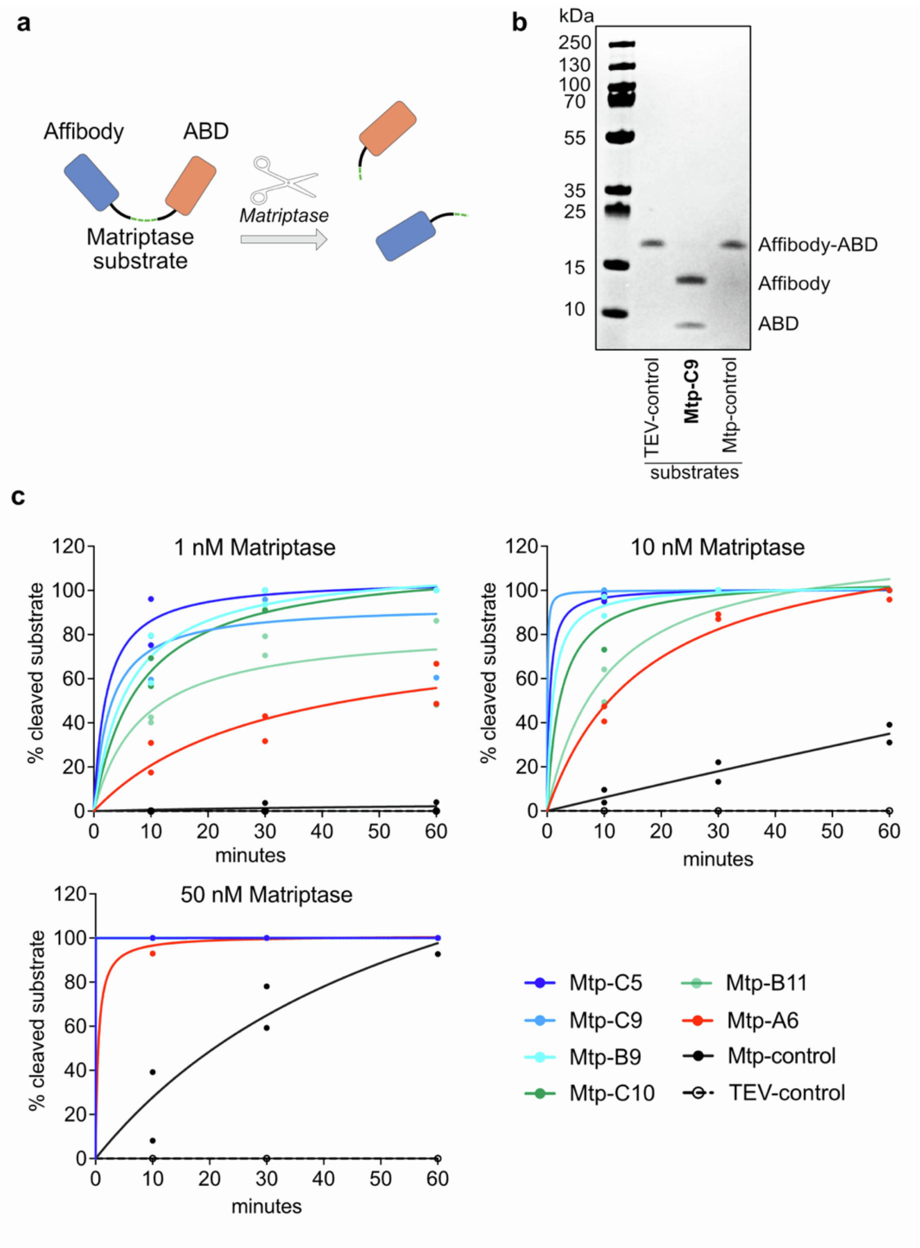

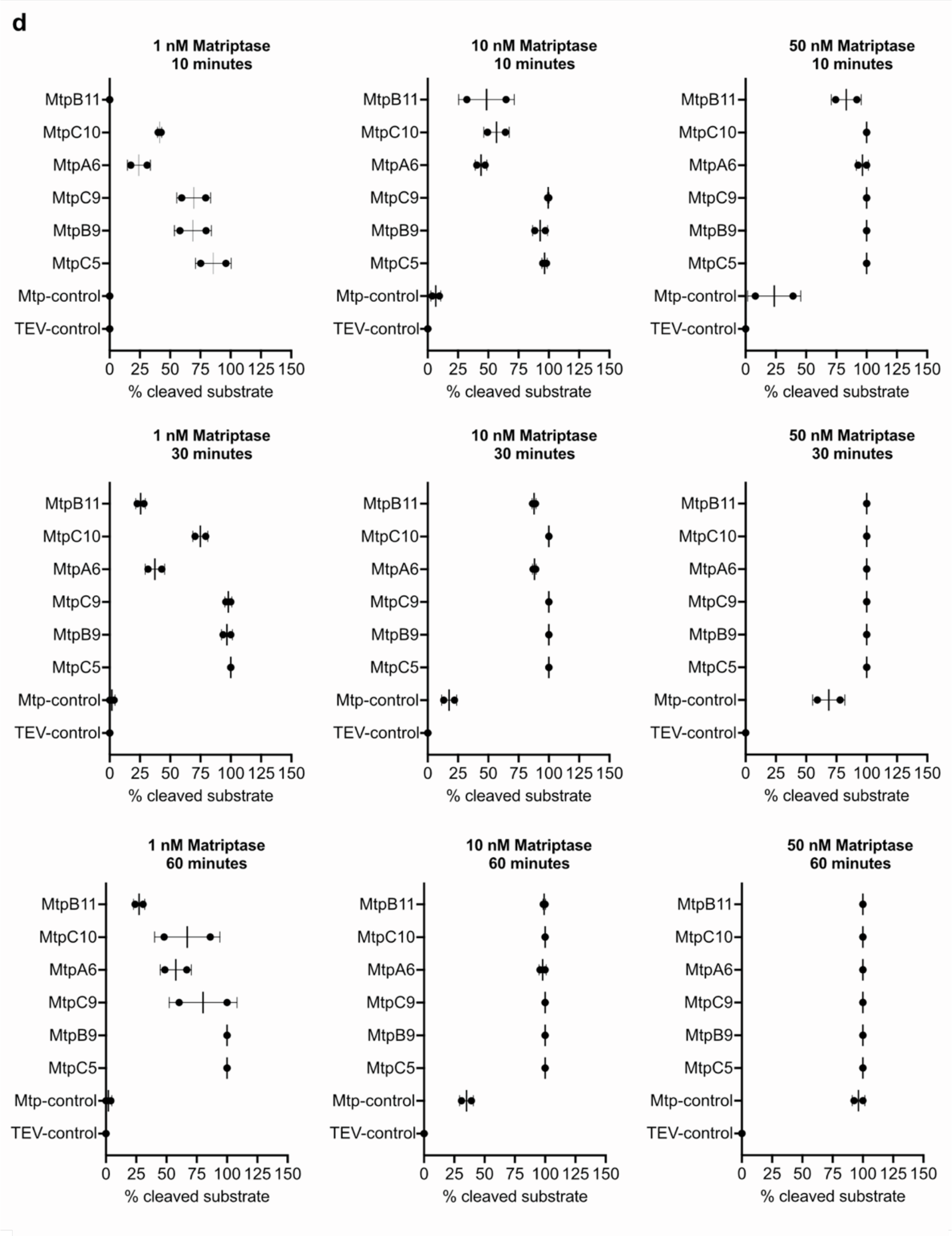
d
1 nM Matriptase 10 minutes
10 nM Matriptase 10 minutes
50 nM Matriptase 10 minutes
1 nM Matriptase 30 minutes
10 nM Matriptase 30 minutes
50 nM Matriptase 30 minutes
1 nM Matriptase 60 minutes
10 nM Matriptase 60 minutes
50 nM Matriptase 60 minutes
MtpB11
MtpC10
MtpA6
MtpC9
MtpB9
MtpC5
Mtp-control
TEV-control
0 25 50 75 100 125 150
% cleaved substrate

Supplementary Table 1. Information of deep sequencing analysis. Related to Figure 2.

Name	n° reads analysed	n° annotated sequences	n° clusters (100% sequence identity)	Cumulative percentage of the 20 largest clusters
Original library	365196	289750	217993	0.06
Sort 1 output	711224	605091	144212	0.2
Sort 2 output	597386	517217	58617	2.8
Sort 3 output	502231	432778	49085	4.5
Sort 4 output	503400	451775	45584	13.7

Supplementary Table 2. Amino acid sequence of the seven substrates selected for their cleavage by matriptase. Related to Figure 2. The positions of the substrate sequence are denoted as P3, P2, P1, P1', P2', and P3', corresponding to the substrate residues surrounding the protease cleavage site.

Name		P3	P2	P1	P1'	P2'	P3'	
MtpC5		V	P	R	G	R	R	
MtpB9		G	A	K	S	R	R	
MtpC9		H	Y	K	G	R	K	
MtpA6		L	K	R	L	M	R	
MtpC10		M	K	R	K	A	R	
MtpC12		P	F	K	R	Q	L	
MtpB11		M	G	R	S	F	Y	
Mtp-control	L	S	G	R	S	D	N	H
